# Structural brain recovery following reductions in adolescent and young adult binge drinking: A longitudinal NCANDA study

**DOI:** 10.1016/j.dcn.2025.101653

**Published:** 2025-12-05

**Authors:** Marybel R. Gonzalez, Ty Brumback, Madison K. Wickershiem, Edith V. Sullivan, Adolf Pfefferbaum, Duncan B. Clark, David B. Goldston, M.J. Meloy, Firas Naber, Eva M. Müller-Oehring, Angelica M. Morales, Fiona C. Baker, Kate B. Nooner, Bonnie J. Nagel, Kilian M. Pohl, Kenneth J. Sher, Sandra A. Brown, Susan F. Tapert, Wesley K. Thompson

**Affiliations:** a Department of Psychiatry and Behavioral Health, The Ohio State University, Columbus, OH; b School of Psychology, Xavier University, Cincinnati, OH; c Department of Psychiatry, University of California San Diego, La Jolla, CA, United States; d Department of Psychiatry and Behavioral Sciences, Stanford University School of Medicine, Stanford, CA, United States; e Center for Health Sciences, SRI International, Menlo Park, CA, United States; f Department of Psychiatry, University of Pittsburgh, Pittsburgh, PA; g Department of Psychiatry and Behavioral Sciences, Duke University School of Medicine, Durham, NC, United States; h Center for Population Neuroscience and Genetics, Laureate Institute for Brain Research, Tulsa, OK, United States; i Department of Neurology and Neurological Sciences, Stanford University, Stanford, CA, United States; j Department of Psychiatry, Oregon Health & Science University, Portland, OR, United States; k Department of Psychology, University of North Carolina Wilmington, Wilmington, NC, United States; l Department of Behavioral Neuroscience, Oregon Health & Science University, Portland, OR, United States; m Department of Psychological Science, University of Missouri, Columbia, MO, United States; n Department of Psychology, University of California San Diego, La Jolla, CA, United States

**Keywords:** Adolescence, Young adults, Alcohol trajectories, Binge drinking, White matter volume, Gray matter volume

## Abstract

Adolescence through young adulthood is a sensitive neurodevelopmental window characterized by ongoing maturation of gray and white matter and heightened vulnerability to alcohol’s neurotoxic effects. Although prior studies link binge drinking with disrupted brain development, the potential for recovery with reduced alcohol use remains underexplored. Using data from 690 participants (ages 12–29) in the National Consortium on Alcohol and NeuroDevelopment in Adolescence to Adulthood (NCANDA-A), we examined the longitudinal impact of binge drinking episodes, and reductions in binge drinking episodes, on regional gray and white matter volumes. Linear mixed-effects models assessed (1) past-year binge drinking frequency, (2) reductions below personal mean binge drinking across time, and (3) transitions in frequency of binge drinking across 10 annual neuroimaging assessments. Results showed that higher binge drinking frequency was associated with decreases in gray matter across frontal, parietal, temporal, and occipital cortices, as well as white matter reductions in frontolimbic and frontostriatal pathways. Reductions below personal mean drinking frequency were also associated with attenuated shrinkage in gray matter volumes. Participants who transitioned from frequent to infrequent binge drinking had significantly larger corpus callosum volumes compared to those with sustained frequent binge episodes. This longitudinal analysis demonstrates consistent negative effects of binge drinking on gray and white matter regions. Importantly, reductions in binge drinking provide evidence for neuroanatomical recovery, particularly in the corpus callosum, and suggest that the degree of recovery may vary by brain region and extent of alcohol use reduction during this key developmental period.

## 1 Introduction

Adolescence through early adulthood, approximately ages 12–29, represents a critical neurodevelopmental period characterized by profound changes across physical, biological, neurocognitive, and social domains. This developmental window is also marked by the initiation and escalation of psychoactive substance use, particularly alcohol (Arnett, 2005). Heightened sensation-seeking and risk-taking behaviors during adolescence (Casey et al., 2008; Steinburg et al., 2008), combined with increased autonomy and expanded social opportunities in emerging adulthood (Arnett, 2020) contribute to elevated vulnerability for alcohol experimentation and misuse.

Alcohol remains the most widely consumed substance across age groups in the United States, with 13 % of 8th graders, 26 % of 10th graders, 42 % of 12th graders, and 84 % 19–30-year-olds reporting any past year alcohol use (Miech et al., 2024). Binge drinking, defined as reaching a blood alcohol concentration (BAC) of 0.08 g/dL or higher, typically consuming five or more drinks for males or four or more for females within about two hours, is particularly concerning due to acute and chronic harms (Kuntsche et al., 2017). Prior research has identified several typical trajectories of binge drinking in adolescents, and for some, this pattern emerges in early to mid-adolescence, peaks in the early twenties, and declines thereafter (Chassin et al., 2002, Maggs and Schulenber, 2005, Schulenberg et al., 2014). Using the National Longitudinal Survey of Youth 1997, Ranker et al., (2023) identified typical and novel binge drinking groups occurring in participants ages 15–30 years of age including “late-escalating” and “high-frequency”. Late-escalators steadily increased their binge drinking’s until plateauing in their late-twenties, while high-frequency drinkers peaked in their mid-twenties, but continued to use at a high rate. Recent data indicate that 28.7 % of young adults aged 18–25 reported binge drinking in the past month (Substance Abuse and Mental Health Services Administration, 2025), with evidence suggesting a rise in binge drinking prevalence among those in their mid-to-late twenties over the last three decades (Leech et al., 2020). Early binge drinking is a robust predictor of subsequent high-intensity alcohol use and related adverse outcomes (Patrick et al., 2024). Excessive alcohol consumption imposes substantial societal costs, playing a causal role in numerous disease, injuries, and other health conditions, as well as being the direct cause for around 2.6 million deaths worldwide (World Health Organization, 2024). Manthey et al., (2021) performed a systemic review and found worldwide the costs of alcohol consumption equate to 1.5–2.6 % of the respective country’s gross domestic product, primarily through productivity loss in the workforce.

Neurodevelopmental trajectories during adolescence and emerging adulthood are marked by asynchronous maturation of gray and white matter. Gray matter volume and cortical thickness typically increase until roughly ages 10–12, decline during adolescence, plateau in the mid-twenties, and decline thereafter, while white matter volume generally increases into the third decade due to ongoing myelination and synaptic pruning (Pfefferbaum et al., 1994, Pfefferbaum et al., 2016, Tamnes et al., 2017, Tapert and Eberson-Shumate, 2022). This maturation sequence proceeds from sensorimotor regions through limbic structures to the prefrontal cortex, which governs higher-order executive functions (Gogtay et al., 2004, Sowell et al., 1999). The developmental imbalance hypothesis posits that earlier maturation of subcortical limbic regions relative to the later-developing prefrontal cortex underlies adolescent risk-taking, including substance use initiation (Casey et al., 2008, Steinburg, 2008). Animal models provide evidence for heightened neuroplasticity and ongoing synaptic refinement during adolescence and early adulthood, rendering the brain to be vulnerable to neurotoxic insults such as alcohol (Spear, 2016). Acute ethanol exposure in still maturing brain regions has been associated with structural and functional alterations, and frontal regions, critical for executive control and behavior regulation, are especially susceptible (Squeglia et al., 2014, De Bellis et al., 2005). Neuroimaging studies in adolescents show dose-dependent associations between alcohol and structural impairment, and individuals who report higher duration and intensity of use result in greater consequences (De Bellis et al., 2005, Lisdahl et al., 2013).

Heavy episodic drinking, or binge drinking, during adolescence through early adulthood has been linked to deviations from normative neurodevelopment, including accelerated declines in gray matter volume (GMV), altered white matter (WM) integrity, and impaired neural connectivity within frontostriatal and frontolimbic circuits (Bava and Tapert, 2010, Luciana et al., 2013, Squeglia et al., 2015). These circuits are crucial for impulse control, decision-making, and emotional regulation, functions often compromised in addiction (Koob and Volkow, 2016). Prior longitudinal studies leveraging data from the National Consortium on Alcohol and NeuroDevelopment in Adolescence (NCANDA) have consistently demonstrated that increases in binge drinking episodes among adolescents exhibit steeper declines in GMV, particularly in frontal and cingulate cortices, compared to non-drinking peers (Pfefferbaum et al., 2018, Infante et al., 2022, Jones et al., 2023). Zhao et al., (2021) examined WM microstructure with DTI which showed heavy drinking during adolescence significantly decreased white matter integrity, most prominently in the anterior and middle corpus callosum. These cortical volume and thinning effects together with white matter volume shrinkage appear related to frequency or intensity of binge drinking (Zhao et al., 2021, Infante et al., 2022).

Owing to the neuroplasticity of the brain, short- and long-term abstinence from alcohol has demonstrated some neurostructural and cognitive improvement (Sullivan and Pfefferbaum, 2019, Winward et al., 2014), though the extent varies, and underlying mechanisms of this recovery are unclear. To date, prior studies looking at the effects of abstinence on the brain have had mixed findings related to the duration and adherence required for recovery (Hanson et al., 2011, Segobin et al., 2014, Zou et al., 2018), and focus primarily on adult populations with a diagnosed alcohol use disorder and/or currently participating in in-patient or out-patient treatment (Demirakca et al., 2011, May et al., 2023). More recently, research has shifted to identify harm reduction approaches, as opposed to complete abstinence, to reduce excessive alcohol use and consequences. Alcohol dependent individuals who continue to consume alcohol at low levels after initiation of treatment (detoxification) were observed to have improvements in gray and white matter volumes comparable to those who remained fully abstinent, but this recovery does not happen synchronously across all regions (May et al., 2023, Meyerhoff and Durazzo, 2020).

Building on this work, our study employs longitudinal neuroimaging and behavioral data to examine within-individual effects of binge drinking episodes and transitions in frequency of use on both gray and white matter volumes, with particular emphasis on frontal, parietal, and temporal brain regions implicated in addiction vulnerability. Our study seeks to replicate previous findings that binge drinking is associated with decreased cortical gray matter volume. We also aim to expand on previous work with a longitudinal dataset that spans a wider developmental window, from early adolescence through emerging and young adulthood (ages 12–29). Assessing neurodevelopmental trajectories up to age 29 not only allows for the consideration of normative brain development that occurs until the mid-20’s (Giedd et al., 1999), but also captures the entire trajectory of binge drinking in adolescence through early adulthood, and “late-escalating” individuals who may not increase their frequency up until their mid-late twenties (Ranker et al., 2023). We include comprehensive analyses of white matter volumes, given their critical role in brain connectivity and cognitive function and their potential susceptibility to alcohol-related neurotoxicity, in addition to gray matter volumes. We hypothesize that less frequent binge drinking would be associated with gray and white matter neurodevelopmental trajectories aligning with no-to-low drinking youth, and that transitions from frequent to less frequent binge drinking will correspond with brain maturation patterns more closely aligned with these healthier trajectories, suggesting the possibility of recovery. Our study aims to better characterize the extent of possible neuroanatomical recovery in gray and white matter regions following a reduction in alcohol consumption levels.

## 2 Methods

### 2.1 Participants

Data were obtained from the National Consortium of Alcohol on Neurodevelopment in Adolescence to Adulthood (NCANDA-A). NCANDA began in 2012 with five United States research sites (University of Pittsburgh, SRI International, Duke University, Oregon Health & Science University, and UC San Diego). In 2013, sites began enrollment of 831 individuals to participate in a baseline visit and nine annual follow-ups. The study used an accelerated longitudinal cohort design with three age bands (12.0–14.9, 15.0–18.9, and 18.0–21.9 years) to capture development from adolescence to early adulthood. Younger ages (12–15) were oversampled at baseline to ensure coverage of critical neurodevelopmental and alcohol initiation periods. To understand how alcohol affects neurodevelopment in adolescence throughout adulthood, by design, participants with no or lower than average drinking frequency were recruited at baseline (n = 690), while the remainder of participants recruited reported a history of binge drinking at baseline and were excluded from the analysis (Brown et al., 2015). A total of n = 454 participants endorsed at least 1 binge drinking episode. Individuals with risk factors for heavy drinking, such as a family history of AUD/SUD, endorsement of ≥ 1 externalizing or ≥ 2 internalizing symptoms, and/or alcohol initiation before age 15, were recruited at higher rates (50 %) to increase the likelihood of later alcohol use. Recruitment efforts included schools, community postings and phone calls in the school catchment areas, and peer referrals. Participants were excluded if they reported any of the following during phone screening: lack of parental availability, MRI contraindications, serious medical conditions, prenatal substance exposure or early developmental problems, current or persistent Axis I psychiatric disorders, or lack of English proficiency (Brown et al., 2015). Each site obtained IRB approval; minors provided written assent with parental consent, and adults provided written informed consent at each annual visit. All sites followed a core protocol at annual visits, including clinical assessments, neuropsychological testing, and neuroimaging, to assess substance use, social and cognitive development, overall functioning, and brain structure and function. Some participants missed follow-up appointments due to various circumstances. Table 1 displays demographic information for the full NCANDA sample at baseline, and the subset included in these analyses. In Supplementary Figure 1, we show the proportion of missing participant data by visit for past year binge episodes.

**Table 1:** Demographics of NCANDA study sample analyzed compared with the complete NCANDA sample, at baseline.

	Sample Analyzed, N (%)	Bingers Only Sample Analyzed, n (%)	Full Sample, n (%)	p-value (n = 690 / n = 454)
Total N	690	454	831	
**Mean Baseline Age,** Years (Range)	15.70 (12.0–21.9)	15.68 (12.0–21.8)	16.17 (12.0 – 21.9)	< 0.001 / < 0.001
**Sex** (F)	348 (50 %)	228 (%)	423 (51 %)	0.90 / 0.86
**Site**				0.86 / 0.42
Site A (%)	93 (14 %)	53 (12 %)	125 (15 %)	
Site B (%)	134 (19 %)	91 (20 %)	166 (20 %)	
Site C (%)	146 (21 %)	91 (20 %)	176 (21 %)	
Site D (%)	136 (20 %)	90 (20 %)	150 (18 %)	
Site E (%)	181 (26 %)	129 (28 %)	214 (26 %)	
**Household Income**				0.50 / 0.21
< $24,999	45 (7 %)	27 (6 %)	57 (7 %)	
$25,000 to $49,999	85 (12 %)	39 (9 %)	95 (11 %)	
$50,000 to $74,999	94 (14 %)	57 (13 %)	102 (12 %)	
$75,000 to $99,999	87 (13 %)	60 (13 %)	106 (13 %)	
$100,000 to $199,999	217 (31 %)	155 (24 %)	266 (32 %)	
> $200,000	130 (19 %)	95 (21 %)	166 (20 %)	
**Parent Education**				0.86 / 0.90
Less than a GED	15 (2 %)	10 (2 %)	15 (2 %)	
Up to high school diploma, GED	36 (5 %)	29 (6 %)	44 (5 %)	
Some college, associate’s degree	91 (13 %)	44 (10 %)	103 (12 %)	
Bachelor’s degree	207 (30 %)	139 (31 %)	248 (30 %)	
Graduate degree	341 (49 %)	232 (51 %)	420 (51 %)	

### 2.2 Binge drinking

**Fig. 1:**
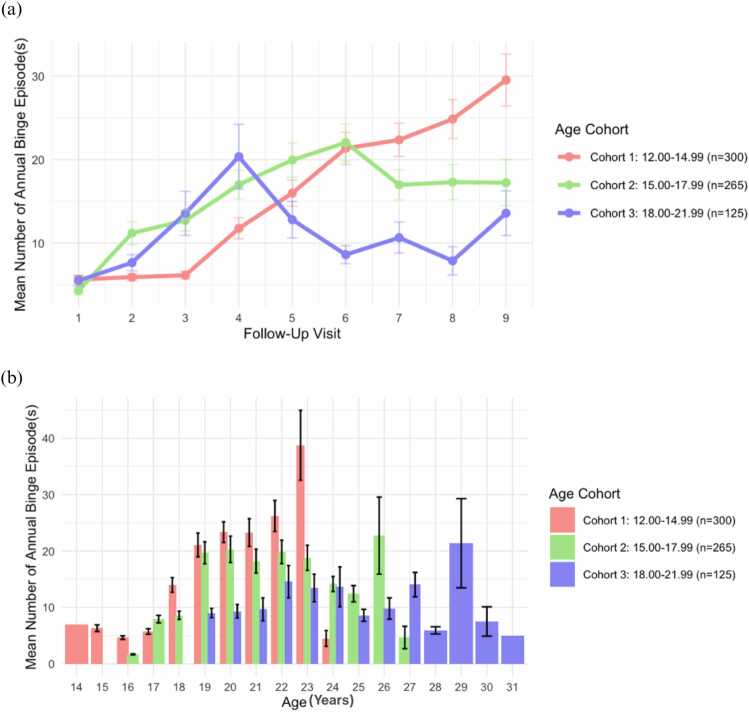
Plots showing (a) linear trajectory of the mean number of past year binge drinking episodes at each annual follow-up visit and (b) by age at baseline for each baseline age cohort.

The Customary Drinking and Drug Use Record (CDDR) (Brown et al., 1998) was collected annually to characterize current and past alcohol/substance use, capturing drinking frequency, average/maximum drinks, and binge drinking frequency. Binge drinking is defined as consuming 4 + drinks for females and 5 + drinks for males on one occasion (National Institute on Alcohol Abuse and Alcoholism, 2007). Participants reporting any binge drinking at baseline were excluded from the current analyses. Binge drinking episodes were assessed as the number of binge drinking episodes in the past year. The mean number of past year binge drinking episodes for each age cohort group across 10 annual follow-up visits is shown in Fig. 1.

### 2.3 MRI data acquisition and analysis

MRI data were drawn from n = 690 NCANDA participants with no or low drinking at baseline who also had multiple usable scans (e.g., no artifacts, entire scan protocol was complete, and scans were of sufficient quality for analysis). All sites used standardized high-resolution structural MRI protocols with 3 T scanners. Three sites (SRI, Duke, UCSD) employed the GE MR750 with an 8-channel head coil and two (UPMC, OHSU) used a Siemens TIM-Trio with a 12-channel head coil. Each site collected data on an ADNI phantom to track scanner quality and variation (Pfefferbaum et al., 2018), facilitating harmonization. T1- and T2-weighted images were acquired in the sagittal plane and processed through a common pipeline that included inhomogeneity correction, and skull stripping, with corresponding intracranial (ICV) defined based on the SRI24 atlas (Pfefferbaum et al., 2018, Rohlfing et al., 2010). From these scans, 42 bilateral Desikan-Killiany regions of interest (ROIs) were derived for analysis, including 34 gray matter ROIs and 8 white matter ROIs.

### 2.4 Statistical analyses

For each set of analyses, 42 linear mixed-effects models (LMEs) were fit using maximum-likelihood estimation, one for each bilateral Desikan-Killiany region of interest (ROIs): 34 with GMV ROIs and 8 with WMV ROIs as the dependent variable. All dependent variables were standardized to have a zero mean and unit standard deviation across all observation. Models testing associations of past-year binge drinking episodes and transition from frequent to infrequent binge drinking were conducted on the sample of N = 690, including non-bingers (i.e., participants with no endorsement of past year binge episodes). The models testing associations of changes in personal mean binge drinking episodes were conducted on the subset of participants who had at least 1 past year binge episode (n = 454).

#### 2.4.1 Past-year binge drinking episodes

In the first set of analyses, the primary independent variable was number of binge drinking episodes in the past year. The variable binge_ij_ (for the *i*th participant at the *j*th visit, for j = 1, …, 9) was the log transformed version of this after first adding one to avoid taking the log of zero, then mean centered at zero (see Infante et al., 2022). Two terms for age were entered in the LME to account for the cohort sequential design of the NCANDA study (Thompson et al., 2011). Baseline age reflected the between-subject variation in age at baseline visits (age_0i_) while within-subject change in age (age_d,ij_) was computed by subtracting age_0i_ from the ith’s participant’s age at visit j. For each LME, binge_ij_, age_0i_, and age_d,ij_ and their 2-way interactions were entered, along with fixed-effect covariates for sex, race, site, and random intercepts for delta age and subject.

#### 2.4.2 Decreases in personal mean binge drinking episodes

We performed a second set of analyses, running 42 LMEs as described above to assess the associations of year-to-year within-person change for binge drinking episodes on GMV and WMV. For each participant separately, we first calculated their mean number of binge episodes (binge_mean_i_) across all visits, accounting for between-participant levels of binge drinking across all visits. For each participant’s visit, we compute binge_mean_d,ij_, or the difference between number of binge episodes in the past year minus binge_mean_i_. The difference score binge_mean_d,ij_ accounts for the within-person change in binge drinking across time (e.g., deviations in binge drinking compared to their personal mean). For each gray and white matter ROI volume, we entered binge_mean_i_, binge_mean_d,ij_, age_0i_, and age_d,ij_ and their 2-way interactions, with fixed covariates of sex, race, site, and random intercepts for delta age and subject. A log-likelihood ratio test compared each model to a null model that controlled for past year binge episodes and the 2-way interactions and covariates described.

#### 2.4.3 Assessment of transitions between frequent to infrequent binge drinking episodes

A 3-level categorical variable characterizing binge drinking transitions using participants’ binge drinking episodes was created by categorizing past year binge episodes into three levels based on the number of binge episodes reported: Frequent (≥24 episodes), Moderate (12–23 episodes), and Infrequent (<12 episodes) (Huang et al., 2006). To examine the transition in binge drinking frequency, past year binge drinking was compared to the previous visit, specifically to capture participants who transitioned from frequent to infrequent drinking (i.e., endorsement of frequent binge drinking in their prior visit to then infrequent binge drinking in their prospective visit). Only data with at least two consecutive visits were included. Then, 42 LMEs tested the association for binge drinking transitions and volume in each brain region by entering binge_ij_, age_0i_, and age_d,ij_ and their 2-way interactions, with covariates for sex, race, site, and random intercepts for delta age and subject. Each model was compared to a null model that controlled for past year binge episodes and the 2-way interactions and covariates described.

#### 2.4.4 Multiple comparisons

Likelihood Ratio Tests (LRT) were conducted to test for significance of all models by comparing each model of interest to the base model described. All reported LRT p-values were 2-sided and Bonferroni-corrected for 42 comparisons (p = 0.05/42 =0.0012).

## 3 Results

### 3.1 Past year binge drinking episodes

**Fig. 2:**
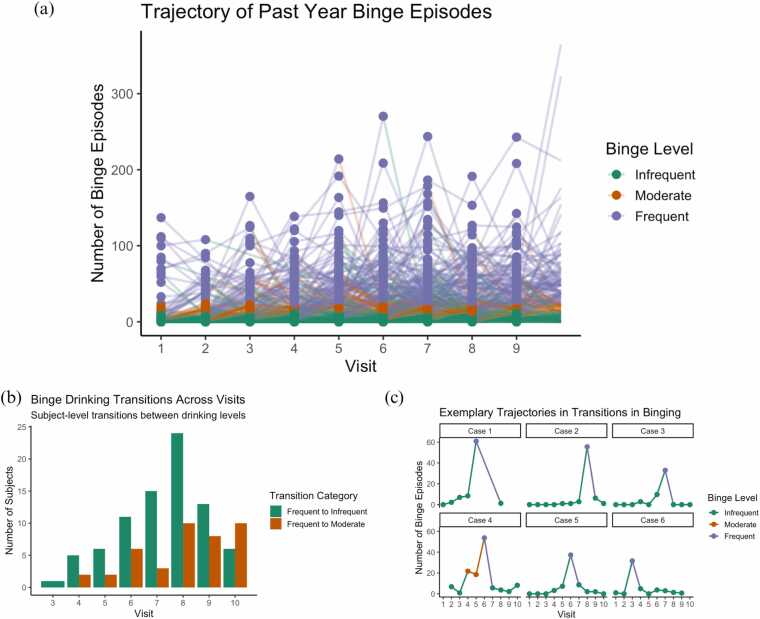
Plots showing the (a) longitudinal trajectories of past year binge drinking severity for infrequent (<12 binges), moderate (12–23 binges), and frequent (24 binges or more) binge drinking of all participants, and (b) the count of total participants in transition groups across visits, and (c) exemplary trajectories for participants who transitioned from infrequent to frequent binge drinking, followed by subsequent decreases.

**Fig. 3:**
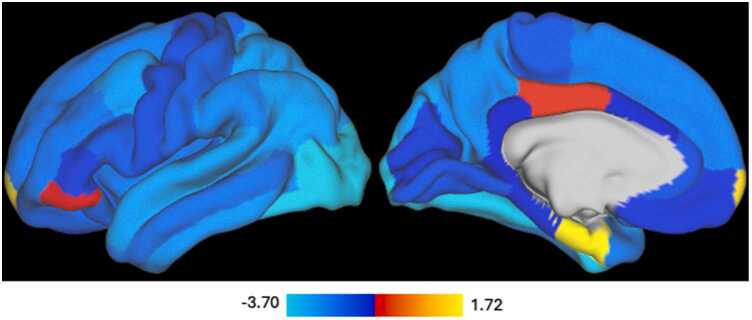
Whole brain effect size map for -logeP value) for the associations of past year binge episodes with grey matter volume for bilaterally averaged Desikan regions. The color coding indicates significant decline in grey matter volume related to greater number of binge drinking episodes, with brighter blue indicating stronger effects.

Fig. 2a shows the distributions of individual binge drinking trajectories across time. Of 42 brain regions tested, 12 grey matter regions and one white matter region remained significantly associated with past year binge drinking episodes after Bonferroni correction. These were three frontal, four parietal, two temporal, one occipital gray matter region, and the corpus callosum, a white matter structure. In each region, more past-year binge drinking episodes was associated with decreased volume. Specifically, higher past year binge drinking was associated with reduced volume in the superior frontal (β = −0.223, p = 0.018) and rostral middle frontal (β = −0.206, p = 0.026) regions, showing accelerated decline in volume across time (interaction p < 0.001 for each region). Whole brain effect sizes (-log_e_(*P* value) for the associations between past year binge drinking episodes and grey matter volume regions are visualized in Fig. 3.

### 3.2 Changes in personal mean binge drinking episodes

The second set of models evaluated whether within-person changes in binge drinking episodes (i.e., deviations from a participant's average binge drinking frequency) were associated with changes in regional brain volumes over time. Using the same Bonferroni-corrected threshold, the three gray matter regions (superior frontal, rostral middle frontal, and caudal middle frontal) and two white matter regions (corpus callosum and its anterior segment) showed significant negative associations with binge drinking trajectories. Notably, increases in binge drinking relative to an individual's average were associated with greater volumetric reductions over time in these regions (e.g., superior frontal: β = −0.013, *p* < 0.001.

### 3.3 Transitions in frequency of binge drinking episodes

The third set of models evaluated whether between-visit transitions in binge drinking frequency were associated with changes in brain volume. In Fig. 2b we show the distribution of transitions between frequent and infrequent use. Fig. 2c shows exemplary cases of individual participant patterns of binge drinking frequency transitions. After Bonferroni corrected thresholds, transitions in binge drinking severity were significantly associated with changes in volume in only one brain region: corpus callosum (LRT p < 0.001; *β* = 0.03, p = 0.02). Specifically, participants who transitioned from frequent to infrequent binge drinking, i.e., between-visit shift from heavy alcohol use (>24 binges) to low use (<12 binges), showed significantly greater white matter volume in the corpus callosum compared to participants with sustained frequent use. The results of all models are reported in Supplementary Table 2. Prior NCANDA investigations have detected brain anomalies among the cohort (Sullivan et al., 2017). We conducted sensitivity analyses excluding the 19 participants with identified brain anomalies, and the findings for all models remained consistent with those reported above. Table 2.

**Table 2:** LME coefficients and p-values for gray matter volume (GMV) and white matter volume (WMV) ROIs. Significant Bonferroni-corrected P values for Likelihood Ratio Tests (LRTs) are bolded.

**Desikan Region**		**Past Year Binge Drinking Episodes**					**Within-Person Change Binge Drinking Episodes**				
		**LRT (P value)**	**log(binge drinking episodes in past year+1)**		**Trajectories: log(binge drinking episodes in past year+1) × within-subject change in age**		**LRT (P value)**	**Binge difference score (past year - mean)**		**Trajectories: Binge difference score x within-subject change in age**	
			***β***	**P value**	***β***	**P value**		***β***	**P value**	***β***	**P value**
**Grey Matter**	**Frontal lobe**										
	Superior frontal	< 0.001	-0.223	0.018	0.012	0.003	< 0.001	-0.013	< 0.001	-0.013	< 0.001
	Rostral middle frontal	< 0.001	-0.206	0.026	0.015	< 0.001	0.021	-0.011	< 0.001	-0.011	< 0.001
	Caudal middle frontal	< 0.001	-0.197	0.005	0.012	< 0.001	< 0.001	-0.009	< 0.001	-0.009	< 0.001
	Pars opercularis	0.0092	-0.104	0.085	0.003	0.231	0.023	-0.006	0.004	-0.006	0.004
	Pars triangularis	0.041	-0.060	0.294	0.006	0.018	0.011	-0.004	0.025	-0.004	0.025
	Pars orbitalis	0.199	0.027	0.814	0.008	0.121	0.386	-0.001	0.771	-0.001	0.771
	Lateral orbitofrontal	0.003	-0.211	0.052	0.013	0.007	0.048	-0.011	0.003	-0.011	0.003
	Medial orbitofrontal	0.4771	-0.117	0.379	0.008	0.148	< 0.001	-0.014	0.004	-0.014	0.004
	Precentral	0.0345	-0.096	0.137	0.005	0.092	< 0.001	-0.006	0.005	-0.006	0.005
	Paracentral	0.0332	-0.152	0.095	0.005	0.253	0.011	-0.006	0.070	-0.006	0.070
	Frontal pole	0.0408	0.045	0.035	0.000	0.621	0.193	0.001	0.059	0.001	0.059
	**Parietal lobe**										
	Superior parietal	< 0.001	-0.337	0.004	0.013	0.009	0.002	-0.014	< 0.001	-0.014	< 0.001
	Inferior parietal	< 0.001	-0.293	0.004	0.018	< 0.001	0.001	-0.013	< 0.001	-0.013	< 0.001
	Supramarginal	< 0.001	-0.256	0.012	0.013	0.003	0.333	-0.009	0.008	-0.009	0.008
	Postcentral	0.005	-0.175	0.058	0.009	0.021	< 0.001	-0.011	< 0.001	-0.011	< 0.001
	Precuneus	< 0.001	-0.190	0.010	0.008	0.009	< 0.001	-0.009	< 0.001	-0.009	< 0.001
	**Temporal lobe**										
	Superior temporal	< 0.001	-0.289	0.003	0.015	< 0.001	0.036	-0.010	0.005	-0.010	0.005
	Middle temporal	0.010	-0.325	0.028	0.019	0.002	0.022	-0.011	0.033	-0.011	0.033
	**Inferior temporal**	< 0.001	-0.275	0.007	0.015	< 0.001	0.055	-0.010	0.004	-0.010	0.004
	**Banks of the superior temporal sulcus**	< 0.001	-0.360	0.004	0.016	0.004	0.558	-0.011	0.010	-0.011	0.010
	**Fusiform**	< 0.001	-0.269	< 0.001	0.009	0.003	0.014	-0.008	< 0.001	-0.008	< 0.001
	Entorhinal	0.015	0.351	0.019	-0.005	0.446	0.375	-0.010	0.005	-0.010	0.005
	Transverse Temporal	0.068	-0.216	0.041	0.004	0.395	< 0.001	-0.007	0.215	-0.007	0.215
	Parahippo-campal	0.071	-0.055	0.521	0.002	0.548	0.074	0.002	0.553	0.002	0.553
	Temporal Pole	0.0013	-0.492	0.017	0.021	0.020	0.412	-0.012	0.091	-0.012	0.091
	**Occipital lobe**										
	**Lateral occipital**	< 0.001	-0.385	< 0.001	0.016	< 0.001	0.026	-0.014	< 0.001	-0.014	< 0.001
	Lingual	0.001	-0.132	0.119	0.002	0.611	0.072	-0.003	0.240	-0.003	0.240
	Cuneus	0.316	-0.062	0.287	0.004	0.137	0.016	-0.004	0.022	-0.004	0.022
	Pericalcarine	0.781	-0.045	0.371	0.002	0.374	0.659	-0.002	0.196	-0.002	0.196
	**Cingulate**						0.118	-0.005	0.018	-0.005	0.018
	Rostral anterior cingulate	0.027	-0.092	0.130	0.007	0.007	0.002	-0.006	< 0.001	-0.006	< 0.001
	Caudal anterior cingulate	0.0013	-0.021	0.669	0.007	< 0.001	0.011	-0.002	0.128	-0.002	0.128
	Posterior cingulate	0.042	0.023	0.606	0.003	0.152	< 0.001	-0.021	< 0.001	-0.021	< 0.001
	Isthmus	0.022	-0.038	0.777	0.014	0.016	0.065	-0.010	0.008	-0.010	0.008
	**Insula**	0.019	-0.259	0.020	-0.005	< 0.001					
**White Matter**	**Corpus Callosum (CC)**	< 0.001	0.090	< 0.001	-0.005	< 0.001	0.160	0.002	0.010	0.002	0.010
	CC Posterior	0.240	0.069	0.292	-0.005	0.053	0.265	0.003	0.203	0.003	0.203
	CC Mid Posterior	0.572	-0.092	0.368	0.001	0.848	0.011	-0.005	0.126	-0.005	0.126
	CC Central	0.043	0	0.997	0.002	0.744	0.002	-0.003	0.443	-0.003	0.443
	CC Mid Anterior	0.031	-0.072	0.392	0.001	0.686	0.561	-0.003	0.369	-0.003	0.369
	CC Anterior	0.023	0.165	0.013	-0.005	0.085	0.048	0.004	0.078	0.004	0.078
	**Cerebellum**	0.630	-0.038	0.076	0.006	0.222	0.466	-0.007	0.101	-0.007	0.101
	**Pons**	0.023	-0.009	0.815	-0.003	0.056	0.569	0.000	0.892	0.000	0.892

## 4 Discussion

This longitudinal study confirms and extends previous findings that binge drinking during adolescence and emerging adulthood is associated with reduced cortical gray matter volume (Pfefferbaum et al., 2018, Infante et al., 2022), particularly in frontal and cingulate regions implicated in executive control and addiction vulnerability. Importantly, by examining a broad developmental window spanning ages 12–29, our results elucidate neurodevelopmental trajectories from early adolescence through young adulthood, a period not well characterized in neuroimaging studies of substance use. In addition to replicating prior work on gray matter alterations (Squeglia et al., 2015, Meda et al., 2017, El Marroun et al., 2021), our inclusion of white matter volume analyses reveals that personal heavy episodic drinking also disrupts brain connectivity substrates critical for cognitive function. Within-subject analyses demonstrate that fluctuations in binge drinking frequency correspond with neurodevelopmental changes, and notably, reductions in binge drinking over time are associated with brain maturation patterns more closely aligned with normative development. Our use of three operationalizations of binge-drinking change allowed us to capture different dimensions of risk and recovery. Past year binge episodes captured recent exposure, whereas within-person change in personal mean episodes captured whether individuals deviated from their own established drinking patterns, an important distinction in patterns of binging. However, the transition from frequent to infrequent binge drinking mapped onto meaningful reductions in high-risk drinking behavior. The observation that participants who transitioned from frequent to infrequent binge drinking exhibited greater white matter in the corpus callosum suggests that reducing binge frequency below high-risk thresholds may have tangible neurobiological benefits.

Our updated results showed robust associations between past-year binge drinking episodes and reduced brain volume across multiple regions. Out of the 42 regions examined, 12 gray matter regions and one white matter region (the corpus callosum) survived Bonferroni correction. The gray matter regions included the superior frontal, rostral middle frontal, and caudal middle frontal cortices; superior and inferior parietal lobules; superior and inferior temporal regions; the lateral occipital cortex; and the fusiform gyrus. In each of these areas, greater binge drinking was associated with smaller volumes, even after accounting for individual differences in baseline age and within-subject age changes. These findings underscore the widespread cortical effects of binge drinking on neurodevelopment, particularly in regions governing cognitive control, sensorimotor integration, and memory.

In the second set of models examining changes in personal mean binge drinking behavior, we found that decreases in binge drinking frequency relative to a participant’s average number of binges were associated with greater volume in the same three frontal gray matter regions (superior frontal, rostral middle frontal, and caudal middle frontal) and two white matter regions (total corpus callosum and its anterior segment). These results suggest that deescalating binge drinking relative to youth’s personal average binge drinking is associated with greater brain volume within regions implicated in executive functioning and interhemispheric communication. Specifically, decreasing binge drinking below your personal mean was associated with increased volume in the superior frontal cortex (β = −0.013, p < 0.001), highlighting the brain’s dynamic sensitivity to fluctuations in alcohol exposure during this period of neurodevelopment.

In a third set of exploratory models, we assessed whether between visit transitions in categories of frequency of binge drinking were associated with preservation or recovery of brain volume. After Bonferroni correction, transitioning from frequent to infrequent binge drinking behavior was associated with greater white matter volume in the corpus callosum compared to participants with sustained frequent binge drinking (β = 0.03, p = 0.02). This finding is the first to provide evidence of the potential for structural recovery in white matter connectivity pathways with reductions in frequency of heavy episodic drinking. In line with these findings, Meyerhoff and Durazzo (2020) and May et al., (2023) found that individuals who adhered to non-abstinent recovery (low-risk drinking) after AUD treatment have regional cortical gray matter volumes similar to those who completely abstained for the study duration. Future studies should confirm these effects and explore whether they generalize to other samples. These findings corroborate and extend those reported by Infante et al. (2022), who documented similar reductions in regional cortical volume associated with adolescent binge drinking. Notably, both studies highlight the vulnerability of the frontal and temporal lobes to alcohol-related neurotoxicity during adolescence and the possibility of structural improvements with reduced exposure.

Together, this body of evidence underscores the importance of interventions specifically targeting binge drinking reduction to support brain health during this critical developmental period. Our results also align with the developmental imbalance hypothesis (Casey et al., 2008) which posits that the asynchronous maturation of subcortical reward systems and the slower-developing prefrontal cortex increases susceptibility to risk-taking during adolescence (Steinburg, 2008, Lees et al., 2021). Alcohol consumption has been associated with oxidative stress, neuroinflammation, and disruption of glutamatergic and dopaminergic signaling. These alcohol neurotoxic effects may selectively impair these already vulnerable frontolimbic circuits. The observed reductions in frontal and interhemispheric white matter volume support this hypothesis and highlight a mechanistic pathway through which binge drinking may impair higher-order cognitive control and are consistent with previous findings on white matter microstructure (Zhao et al., 2021).

The potential for recovery is further supported by a growing body of research demonstrating that reductions in heavy drinking and sustained abstinence can lead to improvements in neurocognitive functioning. Recovery from alcohol-related problems typically involves consistent improvements in alcohol use behavior and related functional domains (Witkiewitz et al., 2020). Prior studies have shown neurocognitive gains after periods of abstinence as brief as four weeks (Winward et al., 2014), and longitudinal follow-ups suggest that even structural brain changes may partially reverse with long-term abstinence in young adults with AUD (Hanson et al., 2011, Pfefferbaum et al., 2014). However, the exact duration and behavioral threshold for structural recovery remain unknown. Our findings, showing attenuated volume loss with reductions in binge drinking and larger corpus callosum volumes among individuals with improved drinking trajectories, add to this evidence base and suggest the possibility of white matter plasticity during emerging adulthood.

Strengths of this study include its accelerated longitudinal design, which allowed us to simultaneously model within- and between-person effects across a wide developmental window; the large, multisite NCANDA sample; and standardized neuroimaging protocols. Our use of linear mixed-effects models with full covariate control and Bonferroni correction bolsters confidence in the robustness of observed effects. Further, incorporating both cortical gray matter and white matter analyses provided a more comprehensive view of how binge drinking affects neurodevelopmental structure and connectivity.

### 4.1 Limitations

Despite these strengths, several limitations should be noted. Our sample was enriched for individuals at high risk for alcohol use, which may limit generalizability. Self-reported binge drinking measures are susceptible to recall bias, although several steps were taken to facilitate recall of binge drinking (e.g., review of social media posts). Future studies could benefit from objective or real-time alcohol monitoring methods. While we assessed structural changes, complementary functional imaging and neuropsychological testing are needed to link these anatomical findings to cognitive, affective, and behavioral outcomes. Scanner and site variability, although accounted for, may introduce noise, and sex-specific effects were not a primary focus of this analysis. A subset of participants included in the analysis had reported brain anomalies (Sullivan et al., 2017), however, sensitivity analyses excluding those participants showed results consistent with those reported here. Although our findings linking decreases in binge drinking with increased corpus callosum white matter suggest a potential for recovery, the extent of true brain structural recovery remains unclear and would require evidence of volume reversal in brain regions previously shown to decrease with a higher number of binge drinking episodes. Of note, while our findings reflect moderate effect sizes, smaller effect sizes for associations of brain-behavior relationships in large samples are to be expected (Owens et al., 2021). Smaller magnitude effect sizes may possibly reflect the multifactorial nature of influences on brain structure as well as the substantial inter-individual variability across participants. While our associations reported here are modest, they replicated prior NCANDA findings and included a longitudinal design, supporting the robustness and meaningful interpretation of our results. Further studies are needed to establish whether de-escalation or cessation of binge drinking results in recovery of brain structure more definitively, and the extent of potential brain structure reorganization.

### 4.2 Public health implications

Given the high prevalence and societal costs of adolescent and emerging adult binge drinking, our findings have meaningful implications for public health. Messaging that links heavy episodic drinking to real-time brain changes may resonate with young people and motivate behavior change. Prevention and intervention efforts aimed at reducing binge drinking, especially those that can prompt early reductions after onset, may have lasting benefits on brain health and cognitive outcomes.

## 5 Conclusion

Our study provides strong evidence that binge drinking during adolescence and emerging adulthood disrupts normative brain development, with both between-person differences and within-person increases in binge drinking linked to cortical and white matter volume reductions. Importantly, reductions in binge drinking were associated with volume preservation, particularly in the corpus callosum, suggesting the possibility of structural recovery. By extending longitudinal neuroimaging analyses across a critical developmental span and incorporating both gray and white matter volume outcomes, this work refines an objective accounting of alcohol’s impact on the developing brain and underscores the importance of early and sustained intervention with the promise of brain structural recovery following drinking reduction.

## CRediT authorship contribution statement

**Sandra A. Brown:** Writing – review & editing, Writing – original draft, Project administration, Funding acquisition, Conceptualization. **Duncan B. Clark:** Writing – review & editing, Project administration, Funding acquisition. **David B. Goldston:** Writing – review & editing, Project administration, Funding acquisition. **Kate B. Nooner:** Writing – review & editing, Project administration, Funding acquisition. **Ty Brumback:** Writing – review & editing, Writing – original draft, Methodology, Formal analysis, Data curation, Conceptualization. **Bonnie J. Nagel:** Writing – review & editing, Project administration, Investigation, Funding acquisition. **Wickershiem Madison K:** Writing – review & editing, Writing – original draft, Visualization, Investigation, Formal analysis. **Kilian M. Pohl:** Writing – review & editing, Visualization, Methodology, Investigation. **Edith V. Sullivan:** Writing – review & editing, Writing – original draft, Project administration, Methodology, Funding acquisition, Conceptualization. **Kenneth J. Sher:** Writing – review & editing, Methodology, Conceptualization. **Adolf Pfefferbaum:** Writing – review & editing, Writing – original draft, Project administration, Funding acquisition, Conceptualization. **Firas Naber:** Writing – review & editing, Writing – original draft, Formal analysis. **Eva M. Müller-Oehring:** Writing – review & editing, Methodology. **Angelica M. Morales:** Writing – review & editing, Project administration, Funding acquisition. **Fiona C. Baker:** Writing – review & editing, Project administration, Methodology, Funding acquisition. **Gonzalez Marybel Robledo:** Writing – review & editing, Writing – original draft, Visualization, Methodology, Formal analysis, Data curation, Conceptualization. **Susan F. Tapert:** Writing – review & editing, Writing – original draft, Project administration, Funding acquisition, Conceptualization. **Wesley K. Thompson:** Writing – review & editing, Writing – original draft, Supervision, Project administration, Methodology, Investigation, Funding acquisition, Formal analysis, Data curation, Conceptualization. **Meloy M. J.:** Writing – review & editing, Writing – original draft, Investigation.

## Declaration of Competing Interest

The authors declare that they have no competing interests.

## Data Availability

Data will be made available on request.

## Footnotes

Appendix A Supplementary data associated with this article can be found in the online version at doi:10.1016/j.dcn.2025.101653.
