# Supplementary material for: Structural brain recovery following reductions in adolescent and young adult binge drinking: A longitudinal NCANDA study
Source: Dev Cogn Neurosci. 2025 Dec 5;77:101653. doi: 10.1016/j.dcn.2025.101653 (PMC12765173; doi:10.1016/j.dcn.2025.101653)
Supplement: Supplementary file 1 — Supplementary material [file mmc1.docx]

**Supplementary Information**

| **Supplementary Table 1**. Comparison of demographics for the NCANDA study sample analyzed compared to the full sample. | | | | |  |
| --- | --- | --- | --- | --- | --- |
|  | Full Study Sample | | Sample Analyzed | | |
|  | N | % of  sample | N | % of  sample | |
| Total N | 831 |  | 690 |  | |
| **Age at baseline (months)** | 16.17  (12.00 21.96) |  | 15.70  (12.00-21.91) |  | |
| **Sex (F)** | 408 | 49.1% | 342 | 49.6% | |
| **Site** |  |  |  |  | |
| Site A (%) | 125 | 15.04% | 93 | 13.48% | |
| Site B (%) | 166 | 19.98% | 134 | 19.42% | |
| Site C (%) | 176 | 21.18% | 146 | 21.16% | |
| Site D (%) | 150 | 18.05% | 136 | 19.71% | |
| Site E (%) | 214 | 25.75% | 181 | 26.23% | |
| ***Household Income*** |  |  |  |  | |
| < $24,999 | 57 | 6.86% | 45 | 6.52% | |
| $25,000 to $49,999 | 95 | 11.43% | 85 | 12.32% | |
| $50,000 to $74,999 | 102 | 12.27% | 94 | 13.62% | |
| $75,000 to $99,999 | 106 | 12.76% | 87 | 12.60% | |
| $100,000 to $199,999 | 266 | 32.01% | 217 | 31.45% | |
| > $200,000 | 166 | 19.98% | 130 | 18.84% | |
| ***Caregiver Education*** |  |  |  |  | |
| Less than a GED | 15 | 1.80% | 15 | 2.17% | |
| Up to high school diploma, GED | 44 | 5.29% | 36 | 5.22% | |
| Some college, associate’s degree | 103 | 12.39% | 91 | 13.19% | |
| Bachelor’s degree | 248 | 29.84% | 207 | 30.00% | |
| Graduate degree | 420 | 50.54% | 341 | 49.42% | |

**Supplementary Table 2**. Results of LMEs for the binge drinking transition models.

|  | | **LRT**  **(P value)** | **Frequent to Infrequent Binging Transition** | | **Frequent to Moderate Binging Transition** | |
| --- | --- | --- | --- | --- | --- | --- |
| **Desikan Regions** | |  | ***β*** | **P value** | ***β*** | **P value** |
| **Grey Matter** | **Frontal lobe** |  |  |  |  |  |
|  | Superior frontal | 0.870 | -0.036 | 0.379 | 0.013 | 0.801 |
|  | Rostral middle frontal | 0.826 | -0.024 | 0.553 | 0.019 | 0.717 |
|  | Caudal middle frontal | 0.869 | -0.026 | 0.3597 | -0.008 | 0.828 |
|  | Pars opercularis | 0.948 | -0.004 | 0.881 | -0.009 | 0.785 |
|  | Pars triangularis | 0.089 | -0.046 | 0.054 | 0.043 | 0.163 |
|  | Pars orbitalis | 0.786 | 0.007 | 0.889 | 0.026 | 0.666 |
|  | Lateral orbitofrontal | 0.672 | -0.039 | 0.407 | 0.045 | 0.465 |
|  | Medial orbitofrontal | 0.926 | 0.002 | 0.977 | 0.049 | 0.481 |
|  | Precentral | 0.348 | -0.035 | 0.191 | 0.038 | 0.270 |
|  | Paracentral | 0.629 | 0.023 | 0.519 | 0.058 | 0.212 |
|  | Frontal pole | 0.378 | -0.008 | 0.254 | -0.010 | 0.269 |
|  | **Parietal lobe** |  |  |  |  |  |
|  | Superior parietal | 0.915 | -0.016 | 0.745 | 0.020 | 0.750 |
|  | Inferior parietal | 0.443 | -0.051 | 0.229 | 0.017 | 0.762 |
|  | Supramarginal | 0.151 | -0.038 | 0.321 | 0.027 | 0.592 |
|  | Postcentral | 0.536 | -0.036 | 0.381 | 0.047 | 0.370 |
|  | Precuneus | 0.612 | -0.038 | 0.177 | -0.002 | 0.966 |
|  | **Temporal lobe** |  |  |  |  |  |
|  | Superior temporal | 0.464 | -0.005 | 0.904 | 0.043 | 0.439 |
|  | Middle temporal | 0.069 | -0.122 | 0.043 | 0.062 | 0.423 |
|  | **Inferior temporal** | 0.209 | -0.034 | 0.401 | 0.055 | 0.286 |
|  | **Banks of the superior temporal sulcus** | 0.166 | -0.106 | 0.025 | -0.031 | 0.609 |
|  | **Fusiform** | 0.206 | -0.067 | 0.026 | 0.001 | 0.9747 |
|  | Entorhinal | 0.383 | 0.004 | 0.952 | 0.093 | 0.240 |
|  | Transverse Temporal | 0.235 | 0.041 | 0.325 | -0.005 | 0.932 |
|  | Parahippocampal | 0.614 | -0.012 | 0.725 | -0.069 | 0.122 |
|  | Temporal Pole | 0.045 | 0.091 | 0.270 | -0.024 | 0.821 |
|  | **Occipital lobe** |  |  |  |  |  |
|  | **Lateral occipital** | 0.019 | 0.111 | 0.044 | -0.050 | 0.106 |
|  | Lingual | 0.265 | 0.017 | 0.688 | -0.006 | 0.789 |
|  | Cuneus | 0.676 | -0.021 | 0.422 | -0.017 | 0.260 |
|  | Pericalcarine | 0.784 | -0.020 | 0.398 | -0.004 | 0.745 |
| **White Matter** | **Cingulate** |  |  |  |  |  |
|  | Rostral anterior cingulate | 0.601 | -0.009 | 0.746 | -0.015 | 0.370 |
|  | Caudal anterior cingulate | 0.189 | -0.034 | 0.147 | -0.024 | 0.065 |
|  | Posterior cingulate | 0.027 | -0.005 | 0.805 | -0.028 | 0.008 |
|  | Isthmus | 0.359 | 0.080 | 0.242 | -0.017 | 0.655 |
|  | **Insula** | 0.558 | -0.022 | 0.680 | -0.05 | 0.095 |
|  | **Corpus Callosum (CC)** | **<0.001** | -0.009 | 0.570 | 0.036 | **<0.001** |
|  | CC Posterior | 0.011 | -0.041 | 0.280 | 0.055 | 0.010 |
|  | CC Mid Posterior | 0.532 | 0.019 | 0.743 | 0.055 | 0.010 |
|  | CC Central | 0.542 | 0.330 | 0.609 | 0.055 | 0.136 |
|  | CC Mid Anterior | 0.537 | -0.074 | 0.141 | -0.031 | 0.274 |
|  | CC Anterior | 0.084 | 0.023 | 0.553 | 0.053 | 0.015 |
|  | **Cerebellum** | 0.783 | 0.005 | 0.944 | 0.010 | 0.784 |
|  | **Pons** | 0.013 | -0.064 | 0.006 | 0.005 | 0.680 |


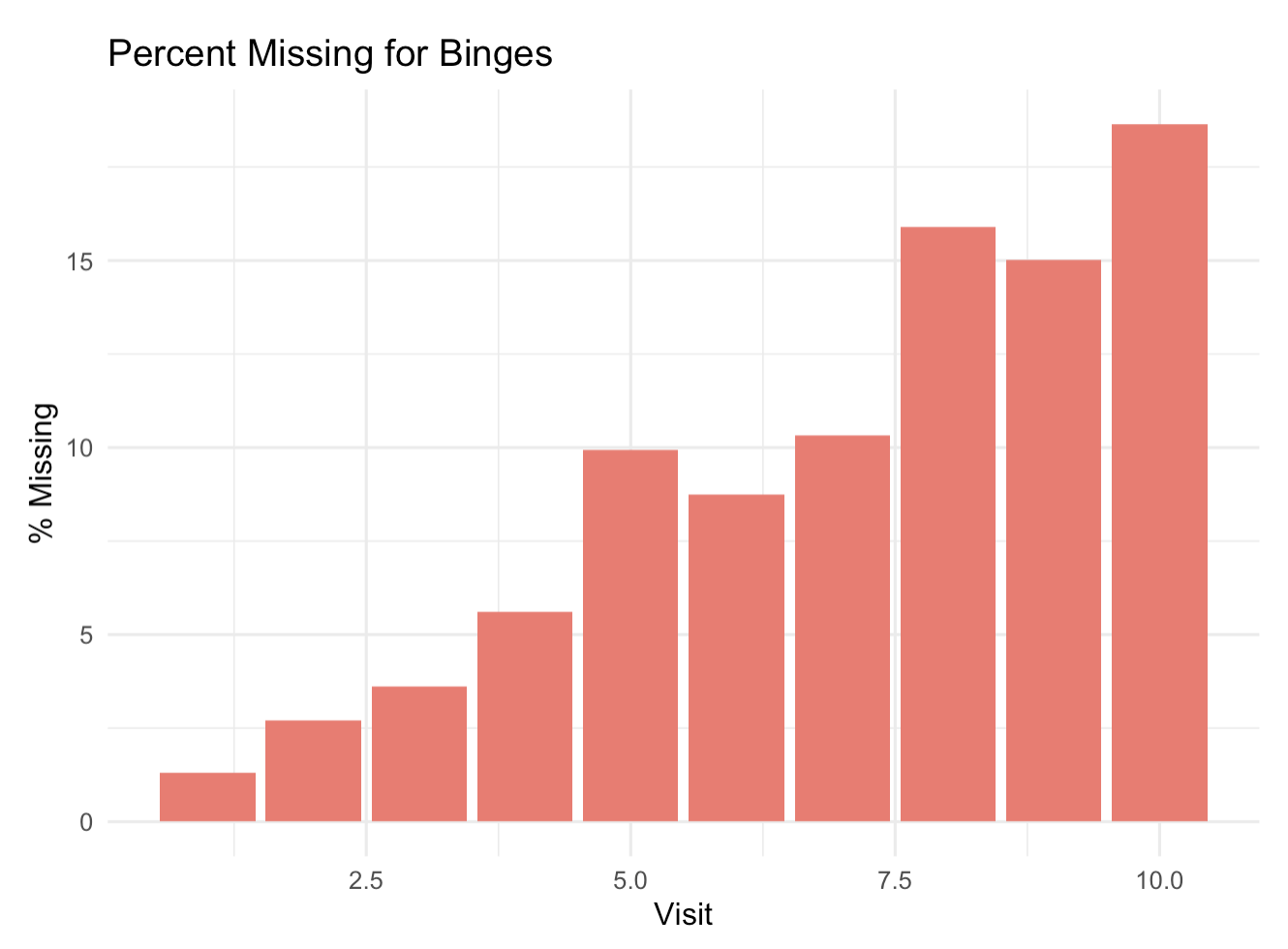


**Supplementary Figure 1.** Percentage of missing data for past year binge episodes by visit in the sample of N = 690 participants.
